# Salinity Stress Response of Rice (*Oryza sativa* L. cv. Luem Pua) Calli and Seedlings

**DOI:** 10.1155/2022/5616683

**Published:** 2022-07-11

**Authors:** Worasitikulya Taratima, Titirat Chomarsa, Pitakpong Maneerattanarungroj

**Affiliations:** ^1^Department of Biology, Faculty of Science, Khon Kaen University, Khon Kaen 40002, Thailand; ^2^Faculty of Veterinary Medicine, Khon Kaen University, Khon Kaen 40002, Thailand

## Abstract

Soil salinity limits plant growth and production. This research investigated a suitable medium for callus induction and plantlet regeneration in the Luem Pua rice cultivar. The effect of salt stress on seedling growth was determined using *in vitro* culture and soil conditions. An efficient protocol for callus induction has been developed by culture sterilized seeds on the Murashige and Skoog (MS, 1962) medium containing 0.5 mg/l benzyladenine (BA) with 1 mg/l 2,4-dichlorophenoxyacetic acid (2,4-D) that resulted in a 100% callus induction. Plantlet regeneration percentage of 49% was recorded on the MS medium containing 4 mg/l BA with 0.5 mg/l 1-naphthaleneacetic acid (NAA) after 4 weeks. For salt stress investigation, the calli were treated on an induction medium containing various concentrations of NaCl (0, 50, 100, 150, and 200 mM), while two-week-old rice seedlings were planted in soil and treated with the same concentration of NaCl for 4 weeks. *In vitro* culture revealed that callus survival percentage decreased when NaCl concentration increased, similar to soil culture. Seedling growth under salinity treatment also decreased when NaCl concentration increased, while other physiological parameters such as total chlorophyll, chlorophyll a, chlorophyll b, green intensity, and chlorophyll fluorescence under light conditions increased under salinity stress. These changes define the growth and physiological salinity tolerance characteristics of Luem Pua rice calli and seedlings. They can be utilized as a baseline for demand-driven *in vitro* rice propagation, providing useful information that can be combined with other agronomic features in rice development or breeding programs to improve the flexibility of abiotic stress-tolerant cultivars.

## 1. Introduction

Rice (*Oryza sativa* L.) is the staple food for more than 50% of the global population [1], especially in Asia [2]. Rice cultivation in Thailand, Indonesia, Myanmar, and Japan totals 30%, while China, India, and Pakistan produce 30%, 21%, and 18% of total world output [3–5]. In dry and semiarid areas, soil salinity is the major environmental constraint limiting plant productivity [6]. Salinity stress, as an important abiotic element, inhibits growth for most plant species [7]. When the electric conductivity (EC) reaches 4 dS m^−1^, the soil is deemed saline (equivalent to 40 mM NaCl), and osmotic pressure of around 0.2 MPa is generated, significantly lowering the yields of most crops [8]. In sensitive species, salt stress inhibits growth and development by reducing leaf area, photosynthesis, respiration rate, protein synthesis, nitrogen fixation, yield, and biomass [9–14].

Rice is considered a glycophytic plant [15], and the most susceptible to salinity among cereal crops. Some rice varieties can tolerate salinity at 3 dS m^−1^. At a salinity of 3.5 dS m^−1^, rice yield decreased by 10%, while at 7.2 dS m^−1^, rice yield decreased by 50% [2]. Salt stress has a negative impact on rice development and yield, which varies according to developmental stages, stress severity level and duration, and variety [16]. Salt stress reduced germination percentage, germination speed, and energy for germination, leading to decreased shoot length, root length, and dry weight in all rice varieties [9]. Rice seedling growth was also inhibited under salinity stress in a physiological and biochemical study [17].

Luem Pua glutinous rice is the staple diet of Hmong Hill tribes in Northern Thailand. This upland area rice is considered to be a drought-tolerant variety. Luem Pua rice is very popular in Thailand due to its high nutritional value, including proteins, vitamins B1 and 2, vitamin *E*, gamma-oryzanol, fatty acids, anthocyanins, omega 3, 6, and 9, zinc, iron, manganese, ascorbic acid, and calcium [18]. Starch products from Luem Pua rice undergo nonenzymatic digestion and can be absorbed within the human small intestine, showing dietary fiber properties. This product is also effective in reducing the size of fat cells in the abdomen, preventing pathology development of the intrathoracic aorta and reducing aorta thickness [19]. Moreover, the delicious taste and the unique variety name have made this rice popular and widely consumed. In Thai, “Luem Pua” means forgetting husband, and maybe wives forget their husbands for a moment while eating this delicious rice. Luem Pua rice has economic potential as a healthy, alternative rice variety, but rice growth and yield are affected by saline soil that is ubiquitous throughout the country, including northeast, central, and coastal areas.

Biotechnological approaches, particularly tissue culture, are now used to attain higher rice quality and yield. *In vitro* propagation in terms of callus culture and adventitious shoot formation is an important tool and fundamental procedure for other advanced biotechnological techniques [20], including crop improvement and preservation aspects [21]. In plant tissue culture, the effects of plant growth regulators (PGRs) have been extensively studied [22–24]. Callus initiation and plant regeneration influenced by PGRs can swiftly produce a large number of plants [23]. During somatic embryogenesis, PGRs play an important role in cell division and differentiation [22], with embryogenic calli required for successful regeneration [23]. Auxins cause embryogenic and organogenic differentiation, cytodifferentiation, and cell division in tissue culture [24]. Among PGRs, the auxin 2,4-D (dichlorophenoxyacetic acid) is well-known for helping to accelerate the proliferation and expansion of embryogenic calli [25]. Many modifications in both type and concentration of PGRs have been evaluated. For rice callus induction, 2,4-D alone or merged with other PGRs such as 1-naphthalene acetic acid (NAA) [26, 27] or kinetin [28] successfully generated calli from seed, while other substances such as casein hydrolysate, proline [22], and coconut water [29] have also been applied for rice callus induction. Natural auxins, including indole-3-acetic acid (IAA), indole-3-butyric acid (IBA), and 1-naphthaleneacetic acid (NAA), primarily interact with cytokinin to promote shoot proliferation and root formation [24]. The interaction of auxin and cytokinin is critical for plant development, and these hormones are routinely used to modulate differentiation in explants *in vitro* plant tissue culture [30]. Numerous reports have been published detailing the combination of auxins and cytokinins in plant tissue culture techniques. Naphthalene acetic acid (NAA) and benzyladenine (BA) have often been used for new plantlet regeneration from rice calli [27, 31, 32]. However, factors such as genotype, type, and concentration of PGRs, physiological and developmental stage of explant, carbon source, medium, and desiccation condition are also considered important parameters affecting new plantlet regeneration from rice calli [24, 27].

Studying physiological reactions at the cellular level is the primary prerequisite before developing a salt-resistant line to overcome the adverse effects of soil salinity, one of the most obstructive influences on crop yield [32]. Callus induction, plantlet regeneration, and the *in vitro* selection of salinity-tolerant Luem Pua rice calli have not yet been investigated. Therefore, here comparative stress resistance was evaluated for cellular tissue (callus) and the organism (seedling) response was observed with salinity assessment of Luem Pua rice growth was conducted under both *in vitro* and *ex vitro* propagation. Findings provide important information for rice cultivation under different salt concentration levels, while basic knowledge from this report can be applied for future rice breeding programs.

## 2. Materials and Methods

### 2.1. Callus Induction

Dehusked seeds were surface sterilized with 70% ethanol for 1 min before shaking for 30 min with 20% (v/v) sodium hypochlorite (Clorox) mixed with 2–3 drops of tween 20 and then washed three times with sterile distilled water

The sterilized seeds were cultured on MS medium with various concentrations of plant growth regulators (PGRs) as 2,4-dichlorophenoxyacetic acid (2,4-D) (0, 1, 1.5, 2 and 2.5 mg/l) and benzyladenine (BA) (0, 0.1, and 0.5 mg/l) for 3 weeks. The cultures were exposed to a light flux density of 40 *μ*mol m^−2^ s^−1^ (16/8 h light/dark) under 25 ± 2°C. Each treatment comprised five replicates, with five calli cultivated in each replicate. Calli derived from the seeds were used as explants in the *in vitro* salinity stress experiment. Callus induction percentage, survival percentage, callus size, fresh weight, and dry weight were recorded. Callus induction percentage was calculated as follows:

(1) $$\begin{array}{c} \text{callus induction percentage}=\frac{\text{final number of seeds with induced calli}}{\text{initial number of seeds}}\times 100. \end{array}$$

Each survived callus was checked using the 2,3,5-triphenyl tetrazolium chloride (TTC) assay according to Towill and Mazur [33], while the response sample was defined as a callus that was still alive and growing or expanding in response to the culture medium. Following Rohmah and Taratima [34], the survival and response percentages were calculated as follows:

(2) $$\begin{array}{c} \text{survival percentage}=\frac{\text{final number of survived calli}}{\text{initial number of calli}}\times 100, \end{array}$$

(3) $$\begin{array}{c} \text{response percentage}=\frac{\text{final number of response calli}}{\text{initial number of calli}}\times 100. \end{array}$$

### 2.2. Plantlet Regeneration

Calli at the same size of 5 mm were cultured on ms medium containing 0, 1, 2, 3, 4, and 5 mg/l BA in combination with 0 and 0.5 mg/l naphthaleneacetic acid (NAA) at 25 ± 2°C with a 16/8 h light/dark cycle under 40 *μ*mol m^−2^ s^−1^ light intensity. Each treatment comprised 10 replicates, with three calli cultivated in each replicate. Regeneration percentage, green spot number per callus, shoot and root number per callus, and survival percentage were investigated after 4 months of culture. The regeneration percentage was calculated as follows:

(4) $$\begin{array}{c} \text{regeneration percentage}=\frac{\text{final number of regenerated calli}}{\text{initial number of calli}}\times 100. \end{array}$$

### 2.3. Salinity Stress under *In Vitro* Propagation

Similar sized calli of 5 mm were used as explants. The calli were treated on an MS medium containing various concentrations of NaCl (0, 50, 100, 150, and 200 mM) and incubated at 25 ± 2°C with a 16/8 h light/dark cycle under 40 *μ*mol m^−2^ s^−1^ light intensity for 4 weeks. The survival rate was determined using the TTC assay and the green spot number per callus was recorded.

### 2.4. Seedling Salinity Stress Treatment

Luem Pua rice seeds were germinated in a Petri dish on filter paper soaked with sterile distilled water for 72 h before transferring into pots (17 cm in diameter).

Each pot was filled with two kilograms of semiloamy clay soil, mixed with peat moss in a 2 : 1 ratio by volume, with four seedlings per pot, and cultivated for 2 weeks. Fourteen-day-old seedlings were used as explants in the salinity stress experiments. Aliquots of 100 ml of NaCl solution at concentrations of 0, 50, 100, 150, and 200 mM were used instead of water every day for 4 weeks. Each treatment was repeated using five replicates with three pots in each replicate.

### 2.5. Growth Performance and Physiological Characteristics

After 4 weeks of seedling salinity treatment, survival rate, plant height, clump no/seedling, leaf number, leaf width, leaf length, green intensity in terms of SPAD unit, chlorophyll a content, chlorophyll b content, total chlorophyll content, chlorophyll fluorescence in light condition (Fv/Fm), and chlorophyll fluorescence in dark condition (Fv'/Fm') were investigated.

Seedling height reduction percentage (SHR%) was calculated according to Islam and Karim [35] as equation (5). The plant height of the control was used as the baseline or denominator to compare the reduced heights of treated seedlings.

(5) $$\begin{array}{c} SHR\%=\frac{\left(\text{plant height at control level}-\text{plant height at saline condition}\right)}{\text{plant height at saline condition}}\times 100. \end{array}$$

The sample was defined as alive when having a green clump and more than two green leaves, while a white or yellow clump with less than two green leaves was identified as a dead plant. The green intensity was recorded using a Chlorophyll Meter (Konica Minolta SPAD-502 Plus), with three areas measured as leaf base, mid leaf, and leaf apex.

Chlorophyll a, chlorophyll b, and total chlorophyll contents were determined. A sample of 0.1 g of mature leaves was ground using a mortar before dissolution in 5 ml of 80% acetone. Another 20 ml of 80% acetone was added once all of the green material had dissolved. The supernatant was detected using a spectrophotometer (Spectronic 20) to measure absorbance at 645 and 663 nm with 80% acetone as a blank. Chlorophyll content was calculated according to Arnon [36] as the following equations:

(6) $$\begin{array}{c} \text{total chlorophyll}\left(\frac{mg}{gtissue}\right)=\left[20.2\left(A645\right)+8.02\left(A663\right)\right]\times \frac{V}{\left(1000\;\times W\right)}. \end{array}$$

(7) $$\begin{array}{c} \text{chlorophyll}a\frac{mg}{g\;tissue}=\left[12.7\left(A663\right)-2.69\left(A645\right)\right]\times \frac{V}{\left(1000\times W\right)}. \end{array}$$

(8) $$\begin{array}{c} \text{chlorophyll}b\frac{mg}{g\;tissue}=\left[22.9\left(A645\right)-4.68\left(A663\right)\right]\times \frac{V}{\left(1000\times W\right)}. \end{array}$$

Here, V is the total volume of solution (ml) and *W* is the weight of leaves (*g*).

Chlorophyll fluorescence in terms of light condition (Fv/Fm units) and dark-adapted leaves (30 min dark) (Fv'/Fm' units) was assessed on mature leaves by a Chlorophyll Fluorometer Handy PEA [37]. All treatments were conducted for four replicates.

### 2.6. Electrical Conductivity (EC_e_)

Soil electrical conductivity was measured following Rayment and Higginson [38]. Every week throughout the NaCl treatments, 3 g of soil samples was collected, placed in 15 ml of deionized water, and allowed to settle for 24 h. Electrical conductivity was measured using a PL-700 Series Bench Top Meter (Gondo: PL-700PC (S)).

### 2.7. Data Analysis

A completely randomized design (crd) was utilized in each treatment for at least three replicates. One-way analysis of variance (one-way ANOVA) was used to examine statistical analysis, while the post hoc test (Duncan's test) was used to compare analyses of mean values at a 95% confidence level. Correlation coefficients between intriguing pairs of growth features at phenotypic levels were used to study growth performance relationships based on Searle [39] and Singh et al. [40] as follows:

(9) $$\begin{array}{c} \text{phenotypic correlation coefficients}\left(rp\right)=\frac{\mathrm{cov}.XP\left(p\right)}{\sqrt{\mathrm{var}.Xp.\mathrm{var}.Yp}}. \end{array}$$

Here, cov.XY (*p*) is the phenotypic covariance between characteristics *X* and Y, and var.X (*p*) and var.Y (*p*) are the variances in phenotypic levels of characteristics *X* and Y, respectively. The SPSS program was used to examine the data.

## 3. Results and Discussion

### 3.1. Callus Induction and Plantlet Regeneration

**Figure 1:**
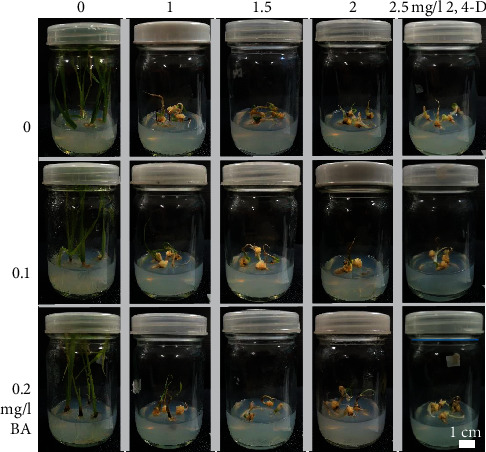
Callus observations of Luem Pua seed cultures on MS medium containing various concentrations of BA and 2,4-D for three weeks.

Light yellow to white calli were formed after 3 weeks of induction on MS medium supplemented with all concentrations of 2,4-D (Table 1 and Figure 1). Seed cultures on a medium without 2,4-D showed seed germination and shoot and root development. Survival percentages of all treatments were not significantly different except for 0.5 mg/l BA with 1.5 and 2.5 mg/l 2,4-D treatments. The highest callus formation percentage (100%) was found in the treatment of 0.5 mg/l BA with 1 mg/l 2,4-D, while 1 mg/l 2,4-D treatment exhibited the highest fresh weight (54.52 mg) and callus length (6.29 mm). Treatment of 0.1 mg/l BA with 2.5 mg/l 2,4-D showed the highest callus width at 4.70 mm, with the highest dry weight (17.12 mg) recorded for the 0.5 mg/l BA with 2 mg/l 2,4-D treatment (Table 1).

**Figure 2:**
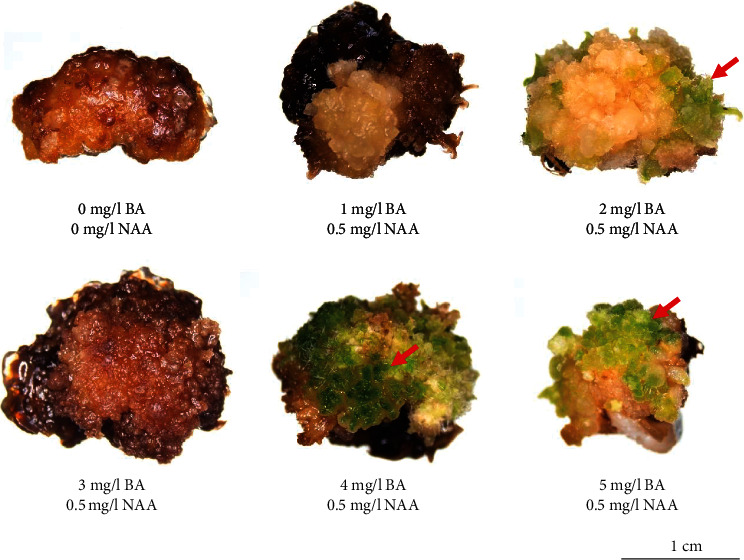
Callus features after culture on MS medium containing various BA and NAA concentrations for four weeks, 0.5 mg/l NAA with 2, 4, and 5 mg/l BA exhibited green spot formation (arrows).

**Figure 3:**
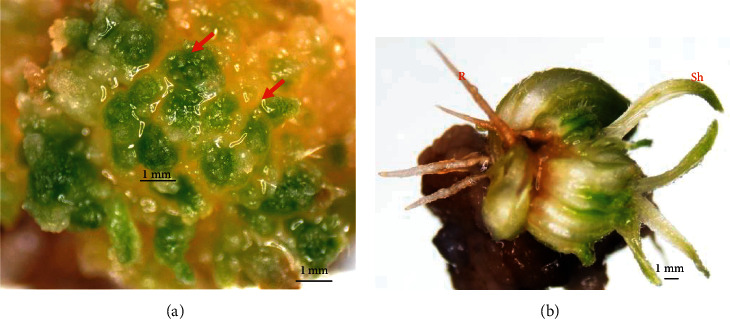
Callus characteristics after culture on MS medium containing 4 mg/l BA with 0.5 mg/l NAA for four weeks. Green spot (arrows) formation on callus (a) and adventitious shoot (Sh) and root (R) derived from callus (b).

Survival percentages and average root numbers per callus of all treatments were not significantly different. All treatments stimulated shoot regeneration from the callus, except for the medium without BA and NAA (Table 2 and Figure 2). Green spot formation was initiated after 2 weeks of culture before developing into new shoots and roots (Figure 3). Some areas of the callus changed from yellow to dark brown after 3-4 weeks of culture. Highest regeneration percentage (49.99%), green spot number per callus (8.7) and shoot number per callus (3.9) were recorded on MS medium containing 4 mg/l BA with 0.5 mg/l NAA (Table 2).

MS medium supplemented with all concentrations of 2,4-D promoted calli formation in Luem Pua rice seed. Previous studies also concurred that appropriate 2,4-D concentration promoted callus formation by encouraging embryogenic capability on the scutellar cells, resulting in proliferation and expansion of rice embryogenic calli [41–44]. New plantlets from calli of Luem Pua rice were regenerated after culture on MS medium with BA and NAA. New adventitious shoots were generated from the calli surfaces. This result also concurred with many previous reports that BA and NAA can be used for plantlet regeneration from rice callus [43, 45, 46]. However, shoot formation in our experiment was more dominant than root formation for high BA : NAA ratios. BA is a plant growth regulator of the cytokinin group, which plays an important role in promoting cell division, abatement of apical dominance, and adventitious shooting [47]. In the presence of auxin, cytokines typically stop rooting because cell division speeds up and impedes differentiation [48]. However, a combination of auxin and various types of cytokinins may be suitable for higher adventitious shoot formation than using only one type of cytokinin. In Topa rice, using 0.5 mg/l NAA in combination with 3 mg/l BA and 0.5 mg/l kinetin gave regeneration percentage of 80% [49]. Optimal conditions for callus induction and regeneration of Luem Pua rice in this study were MS medium containing 0.5 mg/l BA with 1 mg/l 2,4-D and MS medium containing 4 mg/l BA with 0.5 mg/l NAA. However, the success of callus induction or plantlet regeneration depends on many other factors, including type, concentration, and ratio of exogenous plant growth regulators, explant characteristics, and preculture conditions [48, 50].

### 3.2. *In Vitro* Salinity Treatment

**Figure 4:**
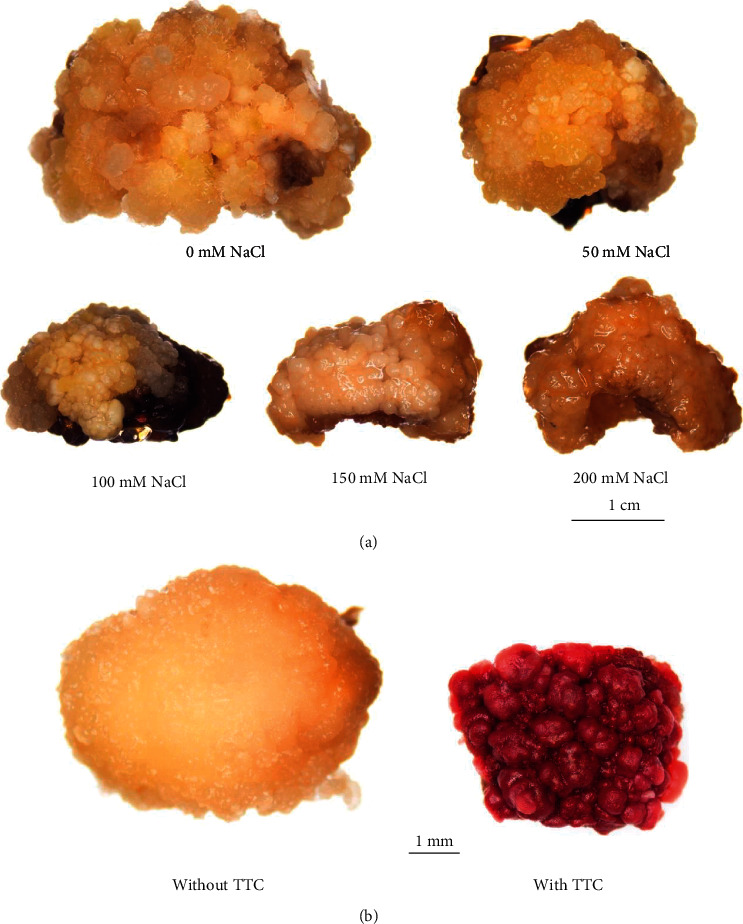
Callus features after culture on MS medium supplemented with 0.5 mg/l BA, 1 mg/l NAA, and various concentrations of NaCl for four weeks (a). Living tissue tested with TTC assay showed red compared to colorless living tissue without TTC assay (b).

All calli showed normal growth during the first week after initiating treatment. After 2 weeks of culture, browning areas formed on all calli, including the control (Figure 4(a)). No regeneration signal was found in this experiment. The survival rate decreased when NaCl concentrations increased. The highest survival percentage was observed in the control group (86.66%), followed by the 50 mM NaCl treatment (76.66%), with no significant difference (Table 3). After 4 weeks of culture, small amounts of green spots formed on the control calli.

Survival rates of all treated calli were determined using the TTC assay. This assay measures the degree of respiration in samples using the enzymatic activity of living plant cells. Colorless TTC is converted to red triphenylformazan by active dehydrogenases in mitochondria [33, 51]. Therefore, living tissue tested under the TTC assay showed red compared to colorless living tissue without TTC assay (Figure 4(b)).

The survival rate of treated calli decreased at high NaCl concentrations, with no green spots or adventitious shoots found. This result differed from studies of IR64 rice [52] and Samba Mahsuri rice [53], where salinity-treated calli of both cultivars showed regeneration performance after treatment with 50 mM NaCl and 75 and 100 mM NaCl in IR64 rice. Luem Pua rice is considered to be a drought tolerance variety; however, our results suggested that this variety may not be tolerant to salinity stress, especially in *in vitro* treatment. *In vitro* systems provide essential tools for stress evaluations, allowing researchers to better understand halophyte plant salt tolerance mechanisms at the cellular or organized tissue level [54]. These studies can also provide information on growth potential and physiological and biochemical responses to NaCl stress at various tissue levels [55]. Therefore, rice callus culture and shoot regeneration responses to salt stress are critical factors in improving rice salt tolerance [56]. Numerous reports about callogenesis and adventitious shoot regeneration of Indica rice have been published, but this investigation focused on the diverse rice cultivar Luem Pua, with an *in vitro* evaluation of calli under various salinity levels.

### 3.3. Seedling Salinity Treatment

**Figure 5:**
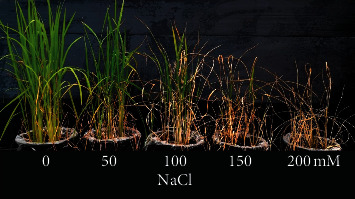
Luem Pua rice phenotypes after salinity treatment for four weeks.

Survival percentage and other growth performance parameters of treated seedlings decreased compared to the control. However, after 4 weeks of treatment, Luem Pua rice seedlings showed tolerance to high salinity levels, with survival percentages of 50 and 100 mM NaCl not significantly different from the control group (Table 4). Seedlings subjected to 200 mM NaCl treatment all died during the third week after treatment (Figure 5 and Table 4). Clump numbers per seedling for all treatments decreased compared to the control, while plant height of 100 and 150 mM NaCl treatments were significantly lower than 50 mM NaCl treatment and the control. Only the 50 mM NaCl treatment exhibited a negative seedling height reduction percentage (SHR%) (−0.01).

Leaf number per seedling, leaf width, and length of treated plants decreased compared to the control, but the green intensity in terms of SPAD unit of 50 and 100 mM NaCl was higher than the control. For chlorophyll content measurement, total chlorophyll, chlorophyll a, and chlorophyll b of 50, 100, and 150 mM NaCl treatments were also higher than the control. The highest chlorophyll b was obtained in the 50 mM NaCl treatment (1.02 mg). Chlorophyll fluorescence values under dark conditions of all treatments were not significantly different from the control, while chlorophyll fluorescence under light-adapted conditions for all treatments was significantly higher than the control (Table 4). The overall growth of NaCl-treated seedlings was not higher than the control, but physiological parameters such as green intensity and chlorophyll content showed improvements.

**Figure 6:**
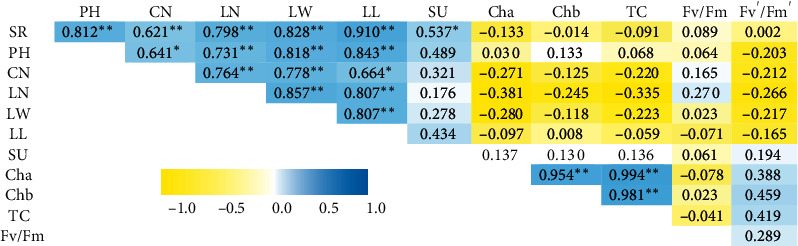
Heat maps explaining the phenotypic correlation coefficient estimation of growth and physiological characteristics in Luem Pua rice after NaCl treatment for four weeks. ^*∗*^Significant correlation at *P* < 0.05; ^*∗∗*^significant correlation at *P* < 0.001. SR = survival rate; PH = plant height; CN = clump no./seedling; LN = leaf number; LW = leaf width; LL = leaf length; SU = SPAD unit; Cha = chlorophyll a; Chb = chlorophyll b; TC = total chlorophyll; Fv/Fm = chlorophyll fluorescence in light condition; Fv'/Fm' = chlorophyll fluorescence in dark condition.

Correlation analyses of growth and physiological traits of treated plants were investigated. Survival rate was highly significantly correlated with plant height, clump number per seedling, leaf number, leaf width, and leaf length (*P* < 0.001). Growth performance in terms of plant height, clump number per seedling, leaf number, leaf width, and leaf length positively correlated with each other, while physiological characteristics such as chlorophyll a, chlorophyll *b*, and total chlorophyll—clump number—negatively correlated with some growth characteristics (Figure 6).

After 4 weeks of salinity treatment, Luem Pua rice seedling growth under salinity level at 1.5 dS m^−1^ decreased (data not shown). This result differed from the rice berry cultivar, where growth increased when exposed to salinity at sodium chloride concentrations up to 8 dS m^−1^ [57]. Increasing concentrations of NaCl decreased plant height, leaf number, leaf width, and leaf length of Luem Pua rice in this study. In other crops such as maize and spinach, growth characteristics such as growth rate, plant height, leaf number, and leaf size were inversely related to NaCl concentration [58]. Salinity stress causes ion toxicity or oxidative stress, imbalance of osmotic stress, stress damage, and cell wall-limited extensibility, which all impact the growth reduction of plants [59]. Soil electrical conductivity also changed after sodium chloride treatment. Salinity levels in soil may depend on other factors and some soil microorganisms also play important roles in soil nutrition balance [60]. High NaCl concentrations in our study did not affect chlorophyll content and chlorophyll fluorescence in both light- and dark-adapted conditions. This result conflicted with Hussain et al. [7], who found that salinity stress reduced photosynthesis parameters in seedlings of Liangyoupeijiu (LYP9) and Nipponbare (NPBA) rice. Salt stress in plants induces free radical formation that destroys the photosynthetic apparatus within the thylakoid membrane, causing chlorophyll to become a colorless substance called chlorophyll bleaching [37]. Green intensity in terms of SPAD unit, total chlorophyll, and chlorophyll a of treated seedlings was higher than the control due to insufficient watering per day of the control approaching the tillering stage with higher growth performance than the treatment. Dehydrated leaves turned pale, and chlorophyll pigment decreased and affected the photosynthesis pathway [61]. Chlorophyll fluorescence measurements of the control and treatments in this study were not significantly different. These values are used to indicate photosystem II (PSII) efficiency [62]. Salinity stress had no effect on the efficiency of PSII in Luem Pua rice. This result differed from sugarcane and cucumber studies, where chlorophyll fluorescence under dark-adapted conditions (*F*_v_/*F*_m_) decreased after salinity treatment resulting in reduced light absorption efficiency, while net photosynthetic rate also reduced [63, 64]. Salinity tolerance cultivars adapt under salt stress by decreasing electrolyte leakage rate, malonaldehyde (MDA), and cumulative proline, which helps to increase salinity tolerance.

Survival rates of NaCl-treated calli and seedlings decreased when NaCl concentrations increased. At high NaCl concentration (200 mM), no seedlings survived (Table 4), while treated calli showed a 30% survival rate (Table 3). Calli under high NaCl concentration displayed salinity stress characteristics at cellular or tissue level, but this did not affect salinity stress tolerance at the organism level as rice seedlings. Calli are composed of unorganized tissue, consisting of undifferentiated parenchymatous cells [65]. NaCl impacted the regeneration performance of treated calli, but many parenchymal cells still survived, while seedlings, as organized tissue, were strongly affected by salinity stress through both physiological and molecular mechanisms such as ionic tolerance, osmotic tolerance, and tissue tolerance [66, 67].

## 4. Conclusions

Embryogenic calli from Luem Pua rice seed were cultured on MS medium containing 0.5 mg/l BA with 1 mg/l 2,4-D, while MS medium containing 4 mg/l BA with 0.5 mg/l NAA was suitable for new plantlet regeneration from calli surfaces. The calli were strongly affected by NaCl. Seedling growth under salinity treatment decreased when NaCl increased, while physiological parameters such as total chlorophyll, chlorophyll a, chlorophyll *b*, green intensity, and chlorophyll fluorescence under light conditions increased under salinity stress. This is the first report on *in vitro* propagation and salinity treatment of Luem Pua rice calli and seedlings. The Luem Pua rice cultivar was found to be sensitive to salinity stress but can grow under low or moderate salinity conditions. Our findings can be utilized to rejuvenate Luem Pua rice seeds, thereby improving the cultivar and also leading to biotechnological development of new varieties by genetic transformation, offering further research avenues on high-yielding abiotic stress-resistant rice cultivars.

## Figures and Tables

**Table 1:** Induction performance and characteristics of calli derived from Luem Pua rice seeds after culture on MS medium supplemented with various concentrations of 2,4-D (dichlorophenoxyacetic acid) and BA (benzylaminopurine) for three weeks.

PGRs		% survival	% callus induction	Growth performance			
BA (mg/l)	2,4-D (mg/l)			Callus fresh weight (mg)	Callus dry weight (mg)	Callus width (mm)	Callus length (mm)
0	0	100 ± 0.00^a^	0 ± 0.00^c^	0.00 ± 0.00^b^	0.00 ± 0.00^b^	0.00 ± 0.00^c^	0.00 ± 0.00^c^
0	1	100 ± 0.00^a^	65 ± 15.00^bc^	54.52 ± 5.37^a^	11.36 ± 1.64^ab^	4.27 ± 0.35^a^	6.29 ± 0.32^ab^
0	1.5	100 ± 0.00^a^	70 ± 5.00^bc^	47.94 ± 7.41^a^	11.02 ± 0.98^ab^	4.09 ± 0.41^ab^	5.59 ± 0.41^bc^
0	2	95 ± 5.00 ^ab^	65 ± 10.00^bc^	14.18 ± 3.00^c^	8.44 ± 1.04^b^	3.75 ± 0.35^ab^	4.79 ± 0.50^bc^
0	2.5	100 ± 0.00^a^	45 ± 9.35^c^	10.32 ± 1.12^c^	6.00 ± 0.39^b^	3.06 ± 0.19^b^	4.48 ± 0.26^bc^
0.1	0	90 ± 6.12^b^	0 ± 0.00^c^	0.00 ± 0.00^c^	0.00 ± 0.00^b^	0.00 ± 0.00^c^	0.00 ± 0.00^c^
0.1	1	100 ± 0.00^a^	75 ± 11.18^bc^	37.46 ± 6.47^ab^	12.54 ± 1.76^ab^	4.07 ± 0.34^ab^	4.94 ± 0.27^bc^
0.1	1.5	100 ± 0.00^a^	80 ± 9.35^bc^	27.02 ± 4.60^ab^	11.30 ± 1.16^ab^	3.74 ± 0.20^ab^	4.30 ± 0.47^c^
0.1	2	95 ± 5.00 ^ab^	70 ± 5.00^bc^	33.68 ± 6.54^ab^	12.24 ± 1.77^ab^	3.84 ± 0.36^ab^	5.61 ± 0.58^bc^
0.1	2.5	100 ± 0.00^a^	70 ± 9.35^bc^	42.42 ± 4.09^a^	12.06 ± 0.66^ab^	4.70 ± 0.24^a^	5.62 ± 0.53^bc^
0.5	0	100 ± 0.00^a^	0 ± 0.00^c^	0.00 ± 0.00^c^	0.00 ± 0.00^b^	0.00 ± 0.00^c^	0.00 ± 0.00^c^
0.5	1	100 ± 0.00^a^	100 ± 0.00^ab^	23.00 ± 4.73^ab^	13.76 ± 1.08^ab^	3.91 ± 0.36^ab^	4.73 ± 0.49^bc^
0.5	1.5	90 ± 6.12^b^	60 ± 12.74^bc^	27.94 ± 4.06^ab^	16.06 ± 1.81^a^	3.90 ± 0.37^ab^	5.12 ± 0.21^bc^
0.5	2	100 ± 0.00^a^	65 ± 10.00^bc^	43.62 ± 5.23^a^	17.12 ± 1.40^a^	4.62 ± 0.23^a^	5.50 ± 0.39^bc^
0.5	2.5	90 ± 6.12^b^	65 ± 10.00^bc^	28.38 ± 7.20^ab^	12.64 ± 1.95^ab^	4.01 ± 0.38^ab^	4.16 ± 0.28^c^

Mean ± SE, *n* = 25. Values followed by different superscripts in the same column are significantly different according to ANOVA and Duncan's Multiple Range Test (*P* < 0.05).

**Table 2:** Regeneration performance of Luem Pua calli after treatment with various concentrations of BA and NAA for four weeks.

MS		% survival	% regeneration	Green spot number/callus	Shoot number/callus	Root number/callus
BA (mg/l)	NAA (mg/l)					
0	0	100 ± 0.00^a^	0.00 ± 0.00^b^	0.00 ± 0.00^b^	0.00 ± 0.00^b^	0.00 ± 0.00^c^
1	0.5	100 ± 0.00^a^	9.99 ± 5.09^b^	0.00 ± 0.00^b^	0.43 ± 0.24^b^	0.00 ± 0.00^c^
2	0.5	100 ± 0.00^a^	13.33 ± 5.44^b^	4.20 ± 2.67^ab^	1.40 ± 0.67^b^	0.13 ± 0.08^a^
3	0.5	100 ± 0.00^a^	3.33 ± 0.33^b^	0.13 ± 0.13^b^	0.06 ± 0.06^b^	0.00 ± 0.00^c^
4	0.5	100 ± 0.00^a^	49.99 ± 11.38^a^	8.70 ± 4.37^a^	3.93 ± 1.17^a^	0.06 ± 0.04^b^
5	0.5	100 ± 0.00^a^	19.99 ± 5.44^ab^	4.63 ± 2.43^ab^	2.43 ± 1.23^ab^	0.00 ± 0.00^c^

Mean ± SE, *n* = 30. Values followed by different superscripts in the same column are significantly different according to ANOVA and Duncan's Multiple Range Test (*P* < 0.05).

**Table 3:** Survival percentage and green spot quantity of salinity-treated calli after four weeks.

NaCl (mM)	% survival	Green spot number/callus
0	86.66 ± 10.18^ab^	0.46 ± 0.24^a^
50	76.66 ± 13.19^ab^	0.00 ± 0.00^b^
100	60.00 ± 16.32^b^	0.00 ± 0.00^b^
150	33.33 ± 14.90^b^	0.00 ± 0.00^b^
200	30.00 ± 15.27^b^	0.00 ± 0.00^b^

Mean ± SE, *n* = 30. Values followed by different superscripts in the same column are significantly different according to ANOVA and Duncan's Multiple Range Test (*P* < 0.05).

**Table 4:** Growth and physiological traits of Luem Pua rice seedling after salinity treatment for four weeks.

Characteristics	NaCl concentration (mM)				
	0	50	100	150	200
% survival	100.00 ± 0.00^a^	100.00 ± 0.00^a^	71.42 ± 10.10^ab^	14.28 ± 0.00^b^	0.00 ± 0.00^b^
Plant height (cm)	50.48 ± 2.68^a^	51.03 ± 1.17^a^	41.83 ± 0.55^ab^	37.55 ± 1.99^b^	—
SHR%	0	−0.01	0.21	0.34	—
Clump no./seedling	3.39 ± 0.08^a^	3.00 ± 0.29^ab^	1.66 ± 0.11^b^	1.75 ± 0.47^b^	—
Leaf no./clump	3.57 ± 0.10^a^	2.89 ± 0.08^a^	2.31 ± 0.10^b^	2.00 ± 0.00^b^	—
Leaf width (cm)	0.59 ± 0.01^a^	0.56 ± 0.01^a^	0.46 ± 0.02^b^	0.43 ± 0.01^b^	—
Leaf length (cm)	25.67 ± 0.75^a^	24.71 ± 0.82^a^	21.24 ± 0.67^ab^	13.21 ± 0.64^b^	—
SPAD unit	22.16 ± 0.71^ab^	26.99 ± 1.23^a^	25.41 ± 1.62^a^	19.22 ± 3.28^b^	—
Chlorophyll a	1.44 ± 0.18^b^	2.02 ± 0.10^a^	1.77 ± 0.15^b^	1.78 ± 0.11^b^	—
Chlorophyll b	0.65 ± 0.09^b^	1.02 ± 0.05^a^	0.78 ± 0.08^b^	0.82 ± 0.06^ab^	—
Total chlorophyll	2.09 + 0.28^b^	3.04 + 0.15^a^	2.55 + 0.23^ab^	2.60 + 0.17^ab^	—
*F*_v_/*F*_m_	0.82 ± 0.01^a^	0.83 ± 0.00^a^	0.81 ± 0.01^a^	0.82 ± 0.00^a^	—
*F*_v_'/*F*_m_'	0.68 ± 0.05^b^	0.79 ± 0.00^a^	0.76 ± 0.02^a^	0.74 ± 0.01^a^	—

Mean ± SE, *n* = 12. Values followed by different superscripts in the same column are significantly different according to ANOVA and Duncan's Multiple Range Test (*P* < 0.05). Chlorophyll measurement unit is mg/g tissue; *F*_v_/*F*_m_ = chlorophyll fluorescence under dark-adapted conditions; *F*_v_'/*F*_m_' = chlorophyll fluorescence under light-adapted conditions.

## Data Availability

The raw data and supplementary information could be obtained from the corresponding author upon request.

## References

[1] Lou W., Wu L., Chen H., Ji Z., Sun Y. (2012). Assessment of rice yield loss due to torrential rain: a case study of Yuhang County, Zhejiang Province, China. *Natural Hazards*.

[2] Hoang T. M. L., Tran T. N., Nguyen T. K. T. (2016). Improvement of salinity stress tolerance in rice: challenges and opportunities. *Agronomy*.

[3] Khush G. S. (2005). What it will take to feed 5.0 billion rice consumers in 2030. *Plant Molecular Biology*.

[4] Calpe C. (2006). *Rice International Commodity Profile*.

[5] Hussain S., Zhang J. H., Zhong C. (2017). Effects of salt stress on rice growth, development characteristics, and the regulating ways: a review. *Journal of Integrative Agriculture*.

[6] Hussain K., Majeed A., Nawaz K., Khizar H. B., Nisar M. F. (2009). Effect of different levels of salinity on growth and ion contents of black seeds (*Nigella sativa* L.). *Current Research Journal of Biological Sciences*.

[7] Hussain S., Cao X., Zhong C. (2018). Sodium chloride stress during early growth stages altered physiological and growth characteristics of rice. *Chilean Journal of Agricultural Research*.

[8] Munns R., Tester M. (2008). Mechanisms of salinity tolerance. *Annual Review of Plant Biology*.

[9] Ologundudu A. F., Adelusi A. A., Akinwale R. O. (2014). Effect of salt stress on germination and growth parameters of rice (*Oryza sativa* L.). *Notulae Scientia Biologicae*.

[10] Farooq M., Hussain M., Wakeel A., Siddique K. H. M. (2015). Salt stress in maize: effects, resistance mechanisms, and management Review. *Agronomy for Sustainable Development*.

[11] Atieno J., Li Y., Langridge P. (2017). Exploring genetic variation for salinity tolerance in chickpea using image-based phenotyping. *Scientific Reports*.

[12] Huang Y., Guan C., Liu Y. (2017). Enhanced growth performance and salinity tolerance in transgenic switchgrass via overexpressing vacuolar Na^+^ (K^+^)/H^+^ antiporter gene (PvNHX1). *Frontiers of Plant Science*.

[13] Zahra N., Mahmood S., Raza Z. A. (2018). Salinity stress on various physiological and biochemical attributes of two distinct maize (*Zea mays* L.) genotypes. *Journal of Plant Nutrition*.

[14] Javaid T., Farooq M. A., Akhtar J., Saqib Z. A., Anwar-ul-Haq M. (2019). Silicon nutrition improves growth of salt-stressed wheat by modulating flows and partitioning of Na^+^, Cl^−^ and mineral ions. *Plant Physiology and Biochemistry*.

[15] Zeng L., Shannon M. C. (2000). Salinity effects on seedling growth and yield components of rice. *Crop Science*.

[16] Zhang R., Hussain S., Wang Y. (2021). Comprehensive evaluation of salt tolerance in rice (*Oryza sativa* L.) germplasm at the germination stage. *Agronomy*.

[17] Liu Y., Wang B., Li J. (2017). Salt response analysis in two rice cultivars at seedling stage. *Acta Physiologiae Plantarum*.

[18] Rice Department of Thailand (2019). Rice Family Thailand [2019-12-03]. https://www.thairicedb.com/index.php.

[19] Praman S., Wanta A., Hawiset T., Sakulsak N., Popluechai S., Somsuan K. (2018). Resistant starch isolated from Luem-Pua glutinous rice decreases adipocyte size of visceral fat and thickness of thoracic aorta in high-fat diet-fed rats. *Chulalongkorn Medical Journal*.

[20] Vennapusa R. A., Vemanna S. R., Reddy B. H. R. (2015). An efficient callus induction and regeneration protocol for a drought tolerant rice indica genotype AC39020. *Journal of Plant Sciences*.

[21] Gosal S. S., Kang M. S. (2012). Plant tissue culture and genetic transformation for crop improvement. *Improving Crop Resistance to Abiotic Stress*.

[22] Kumar S., Singh R., Kalia S., Sharma S. K., Kalia A. K. (2016). Recent advances in understanding the role of growth regulators in plant growth and development *in vitro*-I. conventional growth regulators. *Indian Forester*.

[23] Binte Mostafiz S., Wagiran A. (2018). Efficient callus induction and regeneration in selected indica rice. *Agronomy*.

[24] Abiri R., Maziah M., Shaharuddin N. A. (2017). Enhancing somatic embryogenesis of Malaysian rice cultivar MR219 using adjuvant materials in a high-efficiency protocol. *International journal of Environmental Science and Technology*.

[25] Carsono N., Juwendah E., Liberty L., Sari S., Damayanti F., Rachmadi M. (2021). Optimize 2, 4-D concentration and callus induction time enhance callus proliferation and plant regeneration of three rice genotypes. *Biodiversitas*.

[26] Zuraida A. R., Naziah B., Zamri Z., Sreeramanan S. (2011). Efficient plant regeneration of Malaysian *indica* rice MR 219 and 232 via somatic embryogenesis system. *Acta Physiologiae Plantarum*.

[27] Mohd Din A. R. J., Iliyas Ahmad F., Wagiran A., Abd Samad A., Rahmat Z., Sarmidi M. R. (2016). Improvement of efficient *in vitro* regeneration potential of mature callus induced from Malaysian upland rice seed (*Oryza sativa* cv. Panderas). *Saudi Journal of Biological Sciences*.

[28] Paramasivam S., Ann H. J. (2020). Effect of culture media and conditions on callus induction and plant regeneration of Malaysian wild rice *Oryza rufipogon* IRGC 105491. *Research Journal of Biotechnology*.

[29] Biswas A., Mandal A. B. (2007). Plant regeneration in different genotypes of indica rice. *Indian Journal of Biotechnology*.

[30] Manickavasagam M., Pavan G., Vasudevan V. (2019). A comprehensive study of the hormetic influence of biosynthesized AgNps on regenerating rice calli of indica cv. IR64. *Scientific Reports*.

[31] Chakrabortya A., Hoqueb H., Hasanc N. (2017). Effect of different concentrations of plant growth hormones for *in vitro* regeneration of rice varieties BRRI dhan 28 and BRRI dhan 29. *International Journal of Sciences: Basic and Applied Research*.

[32] Summart J., Thanonkeo P., Panichajakul S., Prathepha P., McManus M. (2010). Effect of salt stress on growth, inorganic ion and proline accumulation in Thai aromatic rice, Khao Dawk Mali 105, callus culture. *African Journal of Biotechnology*.

[33] Towill L. E., Mazur P. (1975). Studies on the reduction of 2, 3, 5-triphenyl tetrazolium chloride as a viability assay for plant tissue cultures. *Canadian Journal of Botany*.

[34] Rohmah K. N., Taratima W. (2021). Effect of chitosan, coconut water and potato extract on protocorm growth and plantlet regeneration of *Cymbidium aloifolium* (L.) sw. *Current Applied Science and Technology*.

[35] Islam M. M., Karim M. A. (1970). Evaluation of rice (*Oryza sativa* L.) genotypes at germination and early seedling stage for their tolerance to salinity. *The Agriculturists*.

[36] Arnon D. I. (1949). Copper enzymes in isolated chloroplasts Polyphenoloxidase in *Beta vulgaris*. *Plant Physiology*.

[37] Theerakulpisut P. (2016). *Plant Physiology under Salt Stress*.

[38] Rayment G. E., Higginson F. R. (1992). Australian Laboratory Handbook of Soil and Water Chemical Methods. *Australian Soil and Land Survey Handbooks*.

[39] Searle S. R. (1961). Phenotypic, genetic and environmental correlations. *Biometrics*.

[40] Singh S. K., Singh V. P., Choudhury D., Dobhal P., Kumar S., Srivastava S. (2018). Estimation of genotypic and phenotypic correlations coefficients for yield related traits of rice under sodic soil. *Asian Journal of Crop Science*.

[41] Gouranga U., Moutushi S., Amitava R. (2015). *In vitro* callus induction and plant regeneration of rice (*Oryza sativa* L.) var. “Sita,” “Rupali” and “Swarna Masuri. *Asian Journal of Plant Science and Research*.

[42] Ho T. L., Te-Chato S., Yenchon S. (2017). Callus induction and plantlet regeneration systems in indica rice (*Oryza sativa* L.) cultivar sangyod. *Walailak Journal of Science and Technology*.

[43] Trunjaruen A., Raso S., Maneerattanarungroj P., Taratima W. (2018). Effects of cultivation media on *in vitro* callus induction and regeneration capabilities of pakaumpuel rice (*Oryza sativa* L.), Thai rice landrace. *Walailak Journal of Science and Technology*.

[44] Mohammed S. (2020). Effects and quantity ranges of some auxins on embryogenic callus induction from upland rice cultivars: an overview. *International Journal of Life Sciences and Biotechnology*.

[45] Rattana K., Theerakulp P., Bunnag S. (2012). The effect of plant growth regulators and organic supplements on callus induction and plant regeneration in rice (*Oryza sativa* L.). *Asian Journal of Plant Sciences*.

[46] Krishnan S. R., Priya A. M., Ramesh M. (2013). Rapid regeneration and ploidy stability of ‘cv IR36’ indica rice (*Oryza sativa* L.) confers efficient protocol for *in vitro* callus organogenesis and *Agrobacterium tumefaciens* mediated transformation. *Botanical Studies*.

[47] Mangena P. (2020). Benzyl adenine in plant tissue culture-succinct analysis of the overall influence in soybean [*Glycine max* (L.) Merrill] seed and shoot culture establishment. *Journal of Biotech Research*.

[48] Moshtaghi N. (2020). Chapter 14 —Tissue and Cell Culture of Saffron Saffron. *Science, Technology and Health Woodhead Publishing Series in Food Science, Technology and Nutrition*.

[49] Jubair T. A., Salma U., Haque N. (2008). Callus induction and regeneration of local rice (*Oryza sativa* L.) variety topa. *Asian Journal of Plant Sciences*.

[50] Schum A., Dohm A. (2003). *Encyclopedia of Rose Science*.

[51] Mikuła A., Niedzielski M., Rybczyski J. J. (2006). The use of TTC reduction asay for assessment of *Gentiana* spp. cell suspension viability after cryopreservation. *Acta Physiologiae Plantarum*.

[52] Priya M. A., Karutha S., Manikandan R. (2011). Effect of NaCl on *in vitro* plant regeneration from embryogenic callus cultures of “cv IR 64” indica rice (*Oryza sativa* L.). *African Journal of Biotechnology*.

[53] Sankepally S. S. R., Talluri V. R., Arulmarianathan J. P., Singh B. (2016). Callus induction and Regeneration capabilities of indica rice cultivars to salt stress. *Journal of Biomolecular Research and Therapeutics*.

[54] Alhasnawi A. N., Zain C. R., Kadhimi A. A., Isahak A., Mohamad A., Yusoff W. M. W. (2017). Accumulation of antioxidants in rice callus (*Oryza sativa* L.) induced by *β*-glucan and salt stress. *Australian Journal of Crop Science*.

[55] Alhasnawi A. N., Kadhimi A. A., Ibrahim A. R. (2014). Salinity tolerant enhancement, tissue culture *in vitro* biochemical procedures. *Journal of Plant Biology Research*.

[56] Rattana K., Bunnag S. (2014). Differential salinity tolerance in calli and shoots of four rice cultivars. *Asian Journal of Crop Science*.

[57] U-bonrat T., Janprasert K., Pongprayoon W. (2017). Physiological responses and clustering of four aromatic rice cultivars to NaCl salt stress. *Burapha Science Journal*.

[58] Tabssum F., Zaman Q. U., Chen Y. (2019). Exogenous application of proline improved salt tolerance in rice through modulation of antioxidant activities. *Pakistan Journal of Agricultural Research*.

[59] Wang Y., Jia D., Guo J., Zhang X., Guo C., Yang Z. (2017). Antioxidant metabolism variation associated with salt tolerance of six maize (*Zea mays* L.) cultivars. *Acta Ecologica Sinica*.

[60] Shilev S. (2020). Plant-growth-promoting bacteria mitigating soil salinity stress in plants. *Applied Sciences*.

[61] Karbaschi M. R., Williams B., Taji A., Mundree S. G. (2016). *Tripogon loliiformis* elicits a rapid physiological and structural response to dehydration for desiccation tolerance. *Functional Plant Biology*.

[62] Guidi L., Lo Piccolo E., Landi M. (2019). Chlorophyll fluorescence, photoinhibition and abiotic stress: does it make any difference the fact to Be a C3 or C4 species?. *Frontiers of Plant Science*.

[63] Cha-um S., Chuencharoen S., Mongkolsiriwatana C., Ashraf M., Kirdmanee C. (2012). Screening sugarcane (*Saccharum* sp.) genotypes for salt tolerance using multivariate cluster analysis. *Plant Cell, Tissue and Organ Culture*.

[64] Zhong M., Wang Y., Zhang Y., Shu S., Sun J., Guo S. (2019). Overexpression of transglutaminase from cucumber in tobacco increases salt tolerance through regulation of photosynthesis. *International Journal of Molecular Sciences*.

[65] Bhatia S. (2015). Plant tissue culture. *Modern Applications of Plant Biotechnology in Pharmaceutical Sciences*.

[66] Roy S. J., Negrão S., Tester M. (2014). Salt resistant crop plants. *Current Opinion in Biotechnology*.

[67] Isayenkov S. V., Maathuis F. J. M. (2019). Plant salinity stress: many unanswered questions remain. *Frontiers of Plant Science*.

